# Deported Men's and Father's Perspective: The Impacts of Family Separation on Children and Families in the U.S.

**DOI:** 10.3389/fpsyt.2020.00148

**Published:** 2020-03-17

**Authors:** Victoria D. Ojeda, Christopher Magana, Jose Luis Burgos, Adriana Carolina Vargas-Ojeda

**Affiliations:** ^1^Department of Family Medicine and Public Health, University of California San Diego School of Medicine, La Jolla, CA, United States; ^2^Department of Medicine, University of California San Diego School of Medicine, La Jolla, CA, United States; ^3^Facultad de Medicina y Psicología, Universidad Autónoma de Baja California, Tijuana, Mexico

**Keywords:** family separation, Mexican migration, deportation, mental health, economic status, immigration enforcement, mixed-methods study, mixed-status family

## Abstract

**Background:** Family separation due to the deportation of a migrant is pervasive, yet less is known about its potential impacts on the social, economic and mental well-being of families remaining in the United States.

**Methods:** We conducted a mixed-methods study. In 2013, 303 Mexican male nationals completed an interviewer-administered questionnaire at a free clinic in Tijuana, Mexico. For this analysis, participants were: (1) ≥18 years; (2) seeking services; (3) Spanish or English speakers and (4) reported a U.S. deportation. Participants answered migration history items and open-ended questions regarding the impact of their deportation on U.S.-based family members. We present descriptive statistics and illustrative quotes for themes identified in the qualitative text data. Using a grounded-theory approach, we considered all data to develop a conceptual framework that others may use to study the consequences of family separation due to deportation.

**Results:** Nearly two-thirds of participants reported living in the U.S. for 11+ years, a similar proportion reported 2+ deportations, and 31% reported being banned from re-entering the U.S. for 11+ years. More than one-half of participants were separated from their nuclear families (spouse/partner and/or children). Deportees who were separated from any family members reported that their families lost income for basic needs (rent/utilities: 50%, food: 44%, clothing: 39%, daycare: 16%, health insurance: 15%); school participation was also negatively impacted (31%). Qualitative data revealed that children ≤18 years remaining in the U.S. experienced mental health symptoms post-parental deportation (i.e., persistent crying, depression, sadness, anger, resentment). Deported fathers consistently expressed frustration at being unable to provide love, care, support, mentorship for their children. Based on our mixed-methods approach, we propose a framework to systematically study the consequences of family separation due to the deportation of fathers.

**Conclusion:** Findings are consistent with the extant research. Binational interventions to support families that experience forced-separation are needed to mitigate short and long-term adverse mental health outcomes, especially among youth in the U.S., and other unfavorable family and household-level outcomes. Funding to understand the implications of maternal deportation and for longitudinal qualitative and quantitative research on migrant-focused interventions and related outcomes is needed.

## Introduction

This study examines the consequences of family separation resulting from the deportation of a male migrant from the U.S. while his family members remain in the U.S. Specifically, we examine how male deportees perceive their deportation impacted the mental health and social and economic security of family members, including their children. Our data were collected in early 2013 and since that time, the term “family separation,” has become closely associated with the Trump administration's policy of separating migrant children from their parents as they enter the U.S. (1–3). However, U.S. immigration policies resulted in the separation of families well before the Trump administration issued its zero tolerance order (4).

Over the last three decades, unauthorized immigration to the United States has been framed as an issue of criminality and a threat to national security (5–7). Immigration policy changes during this period had two synergistic effects that led to the separation of families. First, increased security along the U.S.-Mexico border incentivized migrant workers and their families to settle permanently in the U.S. (8, 9). Next, families became targets of intensified enforcement operations that deported hundreds of thousands of parents, primarily Latino men, separating them from their family members in the United States (10, 11).

During the Clinton administration, the issue of immigration reform became framed within the politics of the War on Crime (4, 6, 7). The White House and Congress supported tougher penalties for unauthorized immigrants, leading to the passage of legislation such as the 1996 Antiterrorism and Effective Death Penalty Act (AEDPA) and the 1996 Illegal Immigration Reform and Immigrant Responsibility Act (IIRIRA) (6). These laws weakened due process and judicial review protections for immigrants facing deportation, expanded the scope of criminal offenses that made immigrants eligible for deportation, mandated the detention of immigrants convicted of certain crimes, expanded cooperation between local law enforcement and federal immigration officers, and created legal frameworks for fast-track deportations (4, 6, 7, 12). In the 15 years after the passage of the AEDPA and IIRIRA >4 million people were deported from the U.S., “more than twice the sum total of every deportation before 1997 (1.9 million people)” (11).

Funding for immigration enforcement steadily rose in the 1990s and early 2000s, and that investment accelerated after the September 11, 2001 (i.e., 9/11) attacks in New York City and Washington, DC (13). Border security became a national security priority and in 2003, immigration enforcement became the purview of the newly formed Department of Homeland Security (5, 12). Enforcement jurisdiction within the United States was given to the Immigration and Customs Enforcement (ICE) agency, one of three DHS agencies that replaced Immigration and Nationalization Services (6–8).

From 1965 to 2017, the majority of unauthorized immigrants in the United States were of Mexican origin (14) due to social, economic and political processes. The Immigration and Nationality Act of 1965 created new restrictions on immigration that disproportionately impacted Mexican migrants; its passage and the simultaneous end of the Bracero Program, eliminated pathways for many Mexican migrant workers to legally enter the United States (15, 16). Despite this “production of illegality,” migration to the United States from Mexico continued to increase in the subsequent decades (16). Relatively relaxed enforcement along with well-established socioeconomic networks, allowed migrant workers to leave their families to engage in circular migration, consisting of finding work in the U.S., sending remittances, building savings, returning to their families in Mexico, and eventually returning to the United States (17). However, intensified border security measures disrupted circular migration flows (17, 18). Because many migrants could no longer reliably return to the United States, they relocated their families to the U.S. (18, 19). The Immigration Reform and Control Act (IRCA) of 1986 further disrupted circular migration networks. The IRCA granted legal amnesty and a pathway to citizenship to millions of undocumented migrants in the U.S., which led “many undocumented migrants who formerly had circulated remained north of the border to claim amnesty and legalize” (20). From 1995 to 2017, the share of the adult unauthorized immigrant population residing in the U.S. for ≥10 years rose from 33 to 66% (14). This increase was highest among unauthorized immigrants of Mexican origin; by 2017, 83% of unauthorized immigrants from Mexico had lived in the U.S. for ≥10 years, more than double the proportion of long-term residents in 2005, while the proportion who had lived in the U.S. for ≤5 years, declined from 34 to 8% over that same period (21). These long-term residents are more likely to have communal and familial ties in the U.S. as well as children who are American citizens by birth (18). In 2018, there were ~5.1 million U.S. citizen-children with at least one parent who was an undocumented immigrant (i.e., mixed-status families) (22).

Weakened legal protections and increased funding for immigration enforcement operations led to unprecedented numbers of deportations in the 2000s and 2010s (4, 23). It is difficult to list a single figure for the total number deportations that occur annually as DHS does not use the term deportation, but instead classifies enforcement actions as either removals or returns (24–26). *Removal* refers to “the compulsory and confirmed movement of an inadmissible or deportable alien out of the United States based on an order of removal” and can include additional criminal penalties and prohibitions from re-entering the U.S. Instead, *returns* do not involve a formal court order nor typically carry additional penalties (24, 27). Enforcement actions classified by DHS as *Removals* better parallel historical definitions and how migrants conceptualize their deportation (28).

During the Obama administration, ~387,000 unauthorized immigrants were removed annually; from 2009 to 2016 a total of 3,094,208 people received a formal deportation order for removal from the U.S. (27). This was more than double the number of formal removals during the Clinton administration and >1 million more than occurred during the George W. Bush administration (27). The large number of mixed-status families paired with intensified enforcement, resulted in the large scale separation of families. One in four deportees is a parent of a U.S. citizen (29), and between 2009 and 2015, ~500,000 parents were deported and separated from their children who remained in the U.S. (30). The present study is situated within this time period.

The Obama administration (2009–2017) implemented policies to limit the impacts of the enforcement strategies on families; during this period, recent arrivals, repeat immigration offenders, and convicted criminals were identified as priorities for enforcement operations (27). However, immigrant rights activists criticized the administration for not doing more to keep families together (31, 32). In 2013, nearly 98% of people deported who reported having U.S. citizen-children were classified by ICE as a priority for removal, and 86% were convicted of a crime (33). How ICE categorizes criminal deportees has also faced scrutiny. In the first quarter of the 2012 fiscal year, only 3.3% of deportation charges filed by ICE were for aggravated felons, 0.01% were for terrorism related charges, while 83.8% were for immigration-only related charges (34). A 2012 TRAC study of ICE deportation filings found that “the vast majority” of adult U.S. citizens would likely be eligible for removal under the Obama administration's enforcement priorities (34). Over 150,000 U.S. citizen-children were separated from a parent due to deportation in 2012 alone (25). In 2013, of the 70,000 parents of U.S. citizen-children who were deported, >10,000 were not convicted of a crime (35). These data reflect conflicts between stated policy goals and the implementation of enforcement actions, resulting in separation of hundreds of thousands of families.

Scholarship on the effects of immigration enforcement on mixed-status families has largely focused on the impact a parent's immigration status and immigration enforcement has on citizen-children. Recent studies have shown that both a parent's unauthorized status (36, 37) and deportation event can negatively impact the quality of life of U.S. citizen-children (38–42). In 2015, the Urban Institute and Migration Policy Institute identified needs and barriers to services faced by citizen-children impacted by parental deportation (33). Investigators found that students whose parent were detained or deported became disengaged from or left school, seeking work to support their families (33). Researchers found that “linguistically and culturally appropriate mental health services” were lacking for citizen-children of deportees (33). A literature review identified the impacts of parental deportation on children between 2009 and 2013 and authors suggested that future research address gaps in the literature by examining how “family separation and loss of parental income affect children's well-being and health and social service needs in the short and long term” (30).

A 2016 mixed-methods study surveyed 48 citizen-children from mixed-status families utilizing the Children's Depression Inventory 2nd Edition scale. They found that 16 of 48 citizen-children scored in the probable depression range; the majority of those with probable depression (*n* = 12 of 16), had a parent who was detained or deported (40). A different 2016 study obtained similar findings when examining post-traumatic stress disorder among 91 Latino U.S. citizen-children from mixed-status families. The study utilized the UCLA Posttraumatic Stress Disorder Reaction Index and found that children whose parents had been detained or deported experienced significantly more potentially traumatic events than children whose parents were legal permanent residents (43). This study also presented a “parent report” including the results of the Behavior Assessment System for Children−2nd Edition, Parent Rating Scales–Child (BASC-2 PRS-C) and the Trauma Symptom Checklist for Young Children—Spanish Version (TSCYC-SP) and found that children of detained or deported parents experienced a greater degree of certain forms of psychological distress, with those children having more internalizing problems (*p* = 0.02), higher measures of depression (*p* = 0.0009) and higher measures of somatization (*p* = 0.04) than the children of legal permanent residents (43).

In the past decade, several studies have also examined the impact immigration enforcement and deportation on mothers remaining with their children in the U.S. after a spouse is deported. These families, particularly those of Mexican descent, were impacted by what Golash-Boza and Hondagneu-Sotelo termed, a “gendered racial removal program” that disproportionately targeted Latino men (11). About 53% of undocumented adults in the U.S. are men, but undocumented men account for >90% of deportees (11). Targeting of Latino men leads to a disproportionate burden and strain on Latina immigrant and citizen spouses who remain in the U.S. (10, 44). For example, partners become single parents who must care for their children without the income and support of their spouse and face challenges finding work to support their families (23, 33). Over 40% of U.S. single-mother households are impoverished, and immigrant women are often barred from utilizing federal welfare programs (45, 46). Many households experience food insecurity (30, 33), which is exacerbated by federal restrictions limiting immigrants' access to Supplemental Nutrition Assistance Program (SNAP) benefits (47) Even those immigrant families that include citizen-children or live in states with food assistance programs, may be afraid to utilize these services (48). Mothers remaining in the U.S. are at an elevated risk of depression and social isolation after a spouse's deportation, which may impair the well-being of children in their care (33). A survey of Latinas in Los Angeles whose spouses were deported found that many lost a vehicle or homes or were forced to move, and older children often fell behind or dropped out of school in order to work to support their family (10).

Gulbas and Zayas conducted a mixed-methods study (37) based on in-depth, semi-structured interviews with 83 citizen-children who had at least one undocumented parent of Mexican origin. Gulbas and Zayas' research describes the impact of immigration enforcement on citizen-children, in what they term the “mixed-status family niche” (37). Access to resources differed between the families in the study based on the “varied assemblages of legal statuses” of members of mixed-status families [e.g., whether both parents were unauthorized or if one parent had legal status; (37)]. Access to resources often determined the extent to which families were impacted post-parental detention or deportation. Gulbas and Zayas developed a “framework for understanding the effects of immigration enforcement on citizen-child outcomes” (37); it draws on ecocultural theories of child development, emphasizing how intrafamilial characteristics are impacted by immigration enforcement, access to resources, and a “cultural script of silence” that prevents discussion of the legal status of parents or other family members or experiences of expulsion from the U.S. (37).

While the literature detailing the experiences of the family members from mixed-status families who remain in the U.S. has grown, fewer studies have examined the experiences and perceptions of deported male fathers who have been separated from their families. Thus, this study relies on data collected in Tijuana, Mexico, a metropolis that borders California. The data remain timely given the persistent efforts of U.S. administrations to target migrant families for deportation (49, 50); findings can inform potential binational policy solutions and the development of evidence-based interventions.

## Methods

### Study Design and Participants

This is a cross-sectional mixed-methods study. In brief, from January to May 2013, a convenience sample of 601 patients attending a free health care clinic in Tijuana's *Zona Norte* [red light district] for structurally vulnerable persons (e.g., homeless, migrants, uninsured), <1 mile of the U.S.-Mexico border, completed an interviewer-administered questionnaire (51). Eligibility criteria were: (1) ≥18 years old; (2) seeking any service; and (3) speaking Spanish or English. This analysis is limited to 303 Mexican male migrants (50% of the full sample) who reported being deported at least once from the United States. All participants provided signed informed consent in accordance with the Declaration of Helsinki, received $10 compensation for their time and refreshments. This study was carried out in accordance with the recommendations of The University of California, San Diego Human Research Protection Program and the Ethics Board of the Clinic.

### Measures

Trained bilingual interviewers administered the survey, lasting ~45 min, via Apple iPad^©^ tablets utilizing Qualtrics survey software (Provo, UT, US). The survey collected quantitative and qualitative data simultaneously via one study instrument. Major domains included: *Socio-demographic* factors included age. *Migration history* included: length of time lived in the U.S. (≤2, 3–5, 6–10, 11–20, 21+ years). *Deportation history* variables included total number of deportations (1, 2–3, 4+), and length of time banned from re-entering the U.S. (not banned from re-entering, ≤5, 6–10 years, 11+years/lifetime). *Communication resources* included: possessed a working Mexican or American cell phone (yes/no), internet café use in the past 6 months (yes/no) and had current access to email (yes/no).

We asked deportees to describe the persons who remained in the U.S. and whom they were separated from as a result of their deportation (partner, kids, parents, siblings, grandparents, other relatives). We then created a variable “separated from nuclear family,” which is defined as being separated from a spouse/partner and/or any children; the comparison group is: “Not separated from nuclear family” defined as a deportee who was separated from extended family (e.g., parents, siblings, grandparents, other relatives) or who lacked any family members in the U.S. We also created a second variable that represented “separated from nuclear family” vs. “separated from extended family” (i.e., parents, siblings, grandparents, or other relatives who remained in the U.S.). Because participants could select multiple responses, individual data may add up to >100%. Additionally, given the sensitive nature of the topics, some data may have been under-reported.

Deportees who were separated from their nuclear family members in the U.S. were asked to identify from a list the economic and other impacts of their deportation on those remaining in the U.S. (loss of income to pay for: rent/utilities, food, clothing, school supplies, health insurance, daycare; need to obtain a new job, need to drop-out of school, need to take in renters, need to move into a new home, need to borrow money for financial obligations of deportee or deportation-related expenses, need to send money to deportee). Participants could select more than one response, therefore the data may exceed >100%.

### Quantitative Data and Analysis

Descriptive statistics for all demographic, migration and deportation history and personal communication variables were generated and stratified according to nuclear family separation (vs. no nuclear family separation/no family separation; Table 1). We also examined the social and economic impacts of deportation, stratifying by nuclear vs. extended family member status; we limited this sub-analysis to deportees who reported having any family members remaining in the U.S. (Table 2). For stratified analyses (Tables 1, 2), we tested for statistical significance between groups using Pearson Chi-square tests for categorical variables.

**Table 1:** Characteristics and migration histories of Mexican males deported from the U.S., stratified by whether they were separated from nuclear family members, Tijuana, Mexico, 2013^c^.

	**Not separated from nuclear family^**a**^ (*n* = 145) %**	**Separated from nuclear Family^**b**^ (*n* = 158) %**	**Full sample of deported migrants (*n* = 303) %**	***p***
**Demographics**				
Age				0.133
18–36	34	24	29	
37–47	39	41	40	
48+	28	35	32	
**Migration history**				
Length of time lived in USA				<0.001
≤2 years	18	1	9	
3–5 years	19	4	11	
6–10 years	22	11	16	
11–20 years	24	41	33	
21+ years	17	43	31	
**Deportation history**				
Total number of deportations				
1	42	28	35	0.050
2–3	35	43	39	
4+	24	29	26	
**Length of time banned from the USA**				
Not banned from returning	40	26	33	0.025
≤5 years	20	19	20	
6–10 years	17	16	16	
11+ years, including lifetime ban	24	38	31	
**Communication resources**				
Has a working Mexican or American Cell phone at time of study	94	91	93	0.263
Used internet café, last 6 months	17	29	23	0.015
Current email access	13	21	17	0.073

a *This category refers to a deported migrant who was separated from extended family (e.g., siblings, parents, grandparents) or lacked any family members in the USA*.

b *A nuclear family is defined as the spouse/partner and/or children*.

c *Percentages may not add to 100% due to rounding*.

**Table 2:** Economic and social impacts of separation on family members remaining in the U.S., as reported by Mexican males deported from the U.S., Tijuana, Mexico, 2013^a^^,^ ^b^.

	**Separated from extended family members^**c**^ (*n* = 69) %**	**Separated from nuclear family^**d**^ (*n* = 158) %**	**Total sample of deported migrants separated from any family members (*n* = 227) %**	***p***
**Loss of income to pay for**^**a**^:				
Rent and utilities	29	59	50	<0.001
Food	22	54	44	<0.001
Clothing	13	50	39	<0.001
School supplies	6	42	31	<0.001
Health insurance	3	20	15	0.001
Daycare	1	22	16	<0.001
Need to obtain new job	10	18	15	0.146
Need to drop-out of school	1	8	6	0.051
Needed to move to a new home	4	10	8	0.148
Need to take renters	1	6	4	0.152
Need to borrow money for deportee	6	6	6	0.878
Need to send money to deportee	22	28	26	0.289

a *Percentages in the table exceed 100% as participants could select multiple responses*.

b *P-values were generated via Chi-Square statistical tests*.

c *This category, Extended Family Members, refers to a deported migrant who was separated from extended family (e.g., siblings, parents, grandparents)*.

d *A nuclear family is defined as the spouse/partner and/or children*.

### Qualitative Text Data

As part of the survey, all deported fathers of children <18 years (*n* = 91) were asked to describe the how their deportation impacted that child who remained in the U.S. (“*In what way has your deportation affected your child or children who are* <*18 years of age?*”). Participants' responses were entered into the survey software verbatim by the interviewers and were brief, ranging from a few words to phrases. For this analysis, we exported the text data into an Excel spreadsheet and responses were coded by three authors (CM, JLB, VO) to identify emergent themes based on the data; conflicts in coding were discussed and resolved. We utilized the methodology of “Coding Consensus, Co-occurrence, and Comparison” which is based on Grounded Theory techniques (52, 53) to generate the codes that underlie our analyses. Some responses were assigned multiple codes. The main themes are described and illustrative quotes are provided in English and Spanish in order to clarify the meaning of the themes. The authors (VO/JLB) translated all quotes into English. Tables 3–**5** provide prevalence estimates and sample sizes for each theme to illustrate its significance (54).

**Table 3:** Themes and illustrative quotes of the mental health impacts of father's deportation on children <18 years remaining in the U.S., as reported by Mexican fathers deported from the U.S., Tijuana, Mexico, 2013.

**Theme**	**Spanish Quotes**	**English translation**
Child's Mental Health is Affected (*n* = 71; 78%)	1. Les ha afectado psicológicamente 2. Mucha tristeza y depresión 3. Lo defino con una palabra—¡trauma! 4. En la separación emocional; les dedicaba tiempo para cuidarlos, jugar y de pronto fui deportado, emocionalmente resultaron afectados	1. It [deportation] has affected them [children] psychologically 2. Much sadness and depression 3. It is defined by one word—trauma! 4. By emotional separation; I dedicated my time to taking care of them, playing, and then suddenly I was deported; they were affected emotionally
Child Feels Sadness & Loneliness (*n* = 28; 31%)	1. Se pusieron tristes, deprimidos, el más chico llora cuando hablo con él, que ya me quiere ver 2. Tristeza porque no ve a su papá 3. Están tristes, no entienden por qué pasó eso 4. Está triste, se pone a llorar, y muchas emociones como ésa 5. La tristeza y distancia 6. Soledad, falta de un padre y una guía	1. They became sad, depressed, the younger one cries when I speak with him; he wants to see me 2. Sadness because he doesn't see his father 3. They are sad, they don't understand why this [deportation] happened 4. He is sad, he cries, and has many emotions like that 5. Sadness and distance 6. Loneliness—the lack of a father and a guide
Child is Experiencing Anger & Resentment (*n* = 8; 9%)	1. A veces mi chavo más grande está enojado conmigo– me extraña 2. …. Otro hijo se puso muy rebelde y trató suicidarse 3. Resentidos conmigo 4. Problemas con mamá aumentan- Mamá se ve mas presionada y los maltrata/grita	1. Sometimes my older kid is angry with me—he misses me 2. My other son became very rebellious and tried to commit suicide 3. They have resentment toward me 4. ….The problems with mom increase and mom is now under more pressure and she treats them badly and yells at them
Child misses deported parent (*n* = 54; 59%)	1. No me miran, preguntan por mí, era una protección que tenían y ahora no puedo ayudarles en nada 2. Mucho porque me extrañan, el sufrimiento de no estar con ellos 3. Por no mirarla y sacarla a pasear me extraña 4. Me extrañan, el cariño del padre… 5. Pues psicológicamente, estaban impuestos a que yo estuviera todos los días, y ahora que no estoy me extrañan	1. They don't see me, they ask about me, [I] was a protection that they had and now I can't help them with anything 2. A lot because they miss me, the suffering of not being with them 3. Because I don't see her and take her on outings—she misses me 4. They miss me—the caring of their father… 5. Well, psychologically, they were accustomed to having me there everyday and now that I'm not there, they miss me
Child Desires Reunification (*n* = 9; 10%)	1. Que no estoy con ellos, nada mas. Quieren que esté con ellos. 2. Que le buscan, que le quieren ver	1. that I'm not there with them, only that—they want me to be with them. 2. they look for him [the deportee] and want to see him

### Conceptual Framework

**Figure 1:**
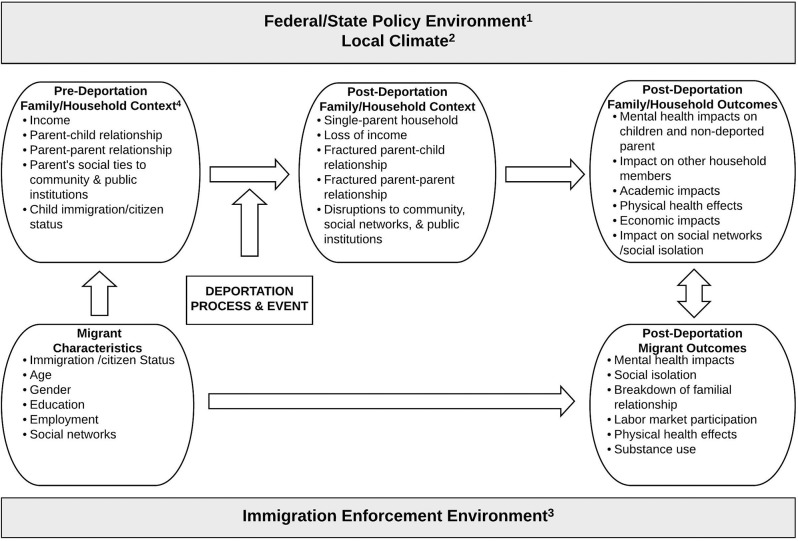
A framework for researching the outcomes of family separation due to paternal deportation. Policy and macro-level factors are shaded in gray. (1) Access to services/programs (welfare programs, food assistance, education, public health insurance, driver's license). (2) Labor market, extended family, schools, religious institutions, food pantries, shelters, child-care centered institutions, stigma/discrimination toward migrants). (3) Removal priorities, sanctuary city/state status. (4) If a two parent household, consider also the second parent's immigration/citizenship status and personal characteristics.

Using a grounded theory approach that draws on the extant literature and the quantitative and qualitative data from this study, we developed an eco-cultural framework to consider the range of potential impacts of paternal deportation on families remaining in the U.S. (Figure 1). Specifically, this framework aims to describe the social, familial, and individual processes/conditions and characteristics that may impact outcomes at the family/household, child, and deportee levels. Our framework was developed following the collection of the data and did not guide the data collection process.

## Results

### Characteristics of Deported Migrants

Our study included 303 Mexican adult deported male migrants (Table 1). The sample was nearly evenly divided by age group, though nearly three-quarters of participants were ages 37+ years. Participants' were established in American communities: 33% reported living in the U.S. for 11–20 years and 31% reported living 21+ years in the U.S.

Participants described their deportation histories; 35% reported only one deportation, 39% reported 2–3 deportations, and 26% reported 4+ deportations. Participants reported whether they were restricted by the U.S. government from re-entering the country; 33% reported not being banned from re-entry, while 20% reported a ban of ≤5 years, 16% reported a ban of 6–10 years, and 31% reported being banned from 11+ years and up-to-a lifetime ban.

Participants described their communication resources; notably, 93% had a working American or Mexican cell phone at the time of the interview, 23% had used an internet café within the prior 6 months, and 17% reported having email access at the time of the study. These were queried as they may have impacted deportees' abilities to communicate with families remaining in the U.S. following their deportation and potentially enhance coping by all parties.

### Characteristics of Deportees Stratified by Separation From Nuclear Family

Table 1 also presents the characteristics of deportees stratified by whether they were separated from their nuclear family vs. participants who were not separated from nuclear family. There were no statistically significant differences in the age distributions of participants. However, the duration of time lived in the U.S. varied significantly by group. For example, those not separated from nuclear family were significantly more likely to be short-term migrants (i.e., ≤2 years; 18 vs. 1% among those separated from the nuclear family; *p* < 0.001). In contrast, those separated from their nuclear family were significantly more likely to be established in the U.S., having lived in the U.S. for 11–20 years (41 vs. 24% among those not separated from nuclear family). Furthermore, those separated from their nuclear family were significantly more likely to have lived in the U.S. for 21+ years than those not separated from their nuclear family (43 vs. 17%, respectively; *p* < 0.001).

We examined deportation histories and access to communication resources, stratifying by group (Table 1). In both groups more than one-half of participants reported being deported more than once. However, those who were not separated from their nuclear family were more likely to not be banned from reentering the U.S. (40 vs. 26% among persons separated from their nuclear family, *p* = 0.025) while those separated from their nuclear family were more likely to report long-term bans (i.e., 11+ years including lifetime bans, 38 vs. 24%, *p* = 0.025). Finally, we examined whether there were variations in deportees' access to communication resources with which they might contact their families in the U.S. We observed no differences by group in the reported access to a working cell phone and email use, however, deportees separated from their nuclear family were more likely to report using an internet café recently (29 vs. 17% among those not separated from nuclear family, respectively, *p* = 0.015).

### Family Members Left Behind

All participants identified which family members they became separated from due to their deportation (data not shown). Nearly half of participants reported being separated from their children (44%) and 38% were separated from their spouse/partner, 38% from siblings, 18% from their parents, 17% from other relatives, and a minority were separated from their grandparents (3%). One quarter of deportees described not being separated from any family members in the U.S. Overall, we determined that 52% of participants were separated from their nuclear families (i.e., spouse/partner &/or children) and 48% were not separated from nuclear family (i.e., extended family and no family members).

### Perceived Economic Impacts of Deportation on Families

Table 2 describes a range of perceived economic impacts on the family left behind in the U.S. resulting from the participants' deportation. In the full sample, about one-third of participants reported their families' losing economic resources to pay rent/utilities (50%) and groceries (44%) while 39% lost income for clothing and school supplies (31%). The deportation also reportedly impacted the families' access to income for health insurance coverage (15%) and day-care (16%). Deportees' also reported that family members were obliged to secure new employment (15%), some abandoned their education as a result of the deportation (6%), or had to move from their residence (8%) or take in renters (4%). Finally, the family left behind reportedly needed to pay for expenses related to the deportees' deportation or other financial obligations (6%) and 26% of families remaining in the U.S. reportedly provided the deportee with money after their deportation.

We stratified the impacts of deportation on families remaining in the U.S. by whether deportees were separated from their nuclear family vs. extended family members (Table 2). Deportees separated from their nuclear family were significantly more likely than those separated from extended family members to report that their nuclear families experienced loss of income for rent/utilities (59 vs. 29%, respectively, *p* < 0.001), food (54 vs. 22%, respectively, *p* < 0.001), clothing (50 vs. 13%, respectively, *p* < 0.001), school supplies (42 vs. 6%, respectively, *p* < 0.001), health insurance (20 vs. 3%, respectively, *p* < 0.001), and day care (22 vs. 1%, respectively, *p* < 0.001); additionally, those who were separated from their nuclear family were more likely to report that someone in the family abandoned their education as a result of the deportation (8 vs. 1%, respectively, *p* = 0.051).

### Perceived Impacts of Deportation on Minor Children Remaining in the U.S.

Deportees' responses (*n* = 91) to the question: “*In what way has your deportation affected your child or children who are* <*18 years of age?*” are described below. Mental health topics were significant emergent themes and these issues clearly constituted key concerns for deported fathers. Illustrative quotes are presented in Tables 3–**5** in English and Spanish, though for brevity, this text presents the English translations.

Table 3 presents themes related to mental health issues. The first theme, “***Child's Mental*** ***Health is Affected*,**” was pervasive in participants' responses and accounted for 71 responses (78%). Notably, participants were acutely aware of and often used broad and non-specific terms to describe the adverse mental health impacts of their deportations on their children, using words such as: “*emotionally*,” “*psychologically*,” “*mentally*.” A prominent sub-theme within the mental health category, was “***Child Feels Sadness & Loneliness”*** (*n* = 28; 31%). Participants described longer-term impacts on their children's affect and emotional state, including persistent sadness, depression, constant crying when communicating with the deported parent, confusion, and feelings of loneliness and isolation. These concepts are represented in Table 3, where the highlighted quotes include: “*They became sad, depressed, the younger one cries when I speak with him; he wants to see me,”* Table 3 provides other supporting quotes. While less commonly discussed by study participants, “***Child is Experiencing Anger and Resentment***” (*n* = 8; 9%, Table 4), was identified by fewer fathers. Some stated that their children were angry and resentful: in a more extreme case “*… my other son became very rebellious and tried to commit suicide.”*

**Table 4:** Themes and illustrative quotes of the father's ability to support children <18 years remaining in the U.S., as reported by Mexican fathers deported from the U.S., Tijuana, Mexico, 2013.

Deported parent cannot help or support child (*n* = 48; 53%)	1. En la separación, no puedo estar con ellos, y no les puedo dar consejos de frente o decirles que los quiero 2. En todo, porque ellos necesitan a su padre en sus vidas 3. La presencia de uno, y la atención que uno les da. Los extraño y me extrañan 4. Pues que no van a tener la figura paterna 5. En que uno no los puede criar, no los puede ver, ni educar 6. Su papi ya no está presente para irlos a ver a sus juegos, para hacer sus tareas, no estoy ahí para guiarlos 7. Porque yo cuidaba a los niños en la tarde y ahora mi ex-esposa tiene que buscar quien cuide a los niños 8. Su hijo menor está deshabilitado…	1. Because of the separation, I can't be there with them, to give them advice face-to-face or tell them that I love them 2. In all aspects, because they need a father in their lives 3. One's presence and the attention that one gives them. I miss them and they miss me. 4. Well, they are not going to have a father figure 5. One can't raise them, can't see them, can't educate them 6. Their daddy is not present to see their [sports] games, to help with homework, I am not there to guide them. 7. Because I used to take care of the kids in the afternoon a and now my ex-wife has to find child care 8. The younger child is disabled

Table 3 presents selected quotes from the second most prevalent theme: “***Child Misses Deported*** ***Parent***” (*n* = 54; 59%). Participants noted that their physical absence was difficult for children and that children missed being with the deported father and being a part of their daily lives and routines. The quotes illustrate an additional role that parents play in their children's' lives, that of protector—physical and emotional guardians of their well-being. The deportation obliviates the possibility of in-person interactions. For example, some responses included: “*They don't see me, they ask about me, [I] was a protection that they had and now I can't help them with anything”* or “*A lot because they miss me; the suffering of not being with them*. A related theme was “***Child Desires Reunification***” (*n* = 9; 10%), wherein children wanted to reunite with the parent and the feeling was reciprocated by the parent; for example: “*that I'm not there with them, only that—they want me to be with them.”*

The third most prevalent theme was “***Deported Parent Cannot Help or Support Child***” which was observed in 48 quotes (53%, Table 4). Deported fathers frequently identified their post-deportation inability to help children in all aspects of their lives. Fathers' remarks convey frustration since they could no longer support their children emotionally and provide them with love, mentorship, advice, caregiving and companionship on a daily basis. These quotes illustrate the instrumental, emotional, and informational support guidance that a parental figure can provide: “*Because of the separation, I can't be there with them, to give them advice face-to-face or tell them that I love them*” or “*Emotionally, they have lost a tutor that can guide them*,”. Children of deportees lose their parental role model: “*Well, they are not going to have a father figure*.”

Regarding “***Adverse Academic Impacts*,**” (*n* = 9; 10%) deportees reported that in some instances, their children's academic performance suffered following the parent's deportation. In one case, a child dropped out of school– “*It [deportation] has affected them a lot—they don't go to school.”* Another parent remarked: “*The studies—they don't want to do things—no hopes/dreams to go on….”* and behavioral changes were also observed at school.

Finally, it was rare for deported parents to identify “**No Impacts of the Deportation”** on their children (*n* = 6, 7%; data not shown). In these instances, parents described having limited relationships or contact with their children, and in one case, the parents withheld information regarding the parent's deportation from the child.

Table 5 also illustrates the negative impacts on families' finances and children's academic trajectories. The first theme, “***Adverse Economic Impacts on Family*”** was reported by one-fifth of participants and demonstrate the impact of paternal deportation on children and the family (*n* = 22; 24%). Consequences ranged from the family needing to relocate their residence and financial challenges associated with paying the rent, to children experiencing deprivation due to insufficient economic resources for basic needs (e.g., clothing). For example, on participant noted: “*They have been deprived of many things, they can barely pay the rent*” and another noted: “*They do not have new shoes, clothes*.” One deportee noted that his wife was forced to have greater labor market participation: “*Economically- because my wife used to work only part-time and now she works full time, 7 days per week.”*

**Table 5:** Themes and illustrative quotes of the adverse economic and academic impacts of father's deportation on children <18 years remaining in the U.S., as reported by Mexican fathers deported from the U.S., Tijuana, Mexico, 2013.

Adverse Economic Impacts on Family (*n* = 22; 24%)	1. Tuvieron que cambiarse de casa…. 2. Se han visto privados de muchas cosas, apenas sacan para la renta 3. En el hecho de que no me ven y en la pérdida económica 4. No tener zapatos nuevos, ropa 5. Lo económico. Porque mi esposa trabajaba puro part- time. Trabajaba solo 5 o 6 horas y 5 días, ahora trabaja 8 horas los 7 días de la semana	1. They had to move to a new house…. 2. They have been deprived of many things, they can barely pay the rent 3. In the fact that they don't see me and in the loss of income 4. They do not have new shoes, clothes 5. Economically- because my wife used to work only part-time and now she works full time 7 days per week
Adverse Academic Impacts (*n* = 9; 10%)	1. No quiere ir a la escuela, no ha rendido lo que antes rendía 2. Los estudios– ya no quieren hacer las cosas; no hopes/dreams goals to go on …. 3. Su papá ya no está presente… para hacer sus tareas, no estoy ahí para guiarlos	1. [child] does not want to go to school and does not perform as he used to 2. The studies—they don't want to do things—no hopes/dreams to go on…. 3. Their daddy is not present to… help with homework, I am not there to guide them

### Conceptual Model: A Framework for Understanding the Outcomes of Family Separation Due to Deportation

Based on both our qualitative and quantitative data and existing research, we developed an overarching conceptual framework of the context and consequences of deportation (see Figure 1); it also considers geographic and policy influences. This socio-ecological framework recognizes that the federal and policy environments shape families' access to public resources (e.g., welfare, nutrition, housing assistance programs, education, health insurance coverage, driver's license). The local community environment also impacts upon families' access to safety-net resources and social networks, such as the employment and business development opportunities, access to food pantries, shelters, religious and child-centered institutions (e.g., daycare, after school programs, enrichment programs), and extended family. Community level stigma toward migrants may also adversely impact individuals' mental health (37, 55). The central section addresses pre-deportation sociodemographic and immigration and citizenship characteristics of the migrants, including parents, which impact upon the characteristics of the pre-deportation household and family structure. Notably, as the immigration policy and enforcement environments change, the type, frequency and impacts of the deportation events are understood to vary (e.g., deportation policy during the Obama administration focused on “criminals” vs. deportation policy under the Trump administration which in 2019 was stated to include “collateral” deportations of non-targeted migrants) (27, 50). Importantly, local factors also impact upon immigration enforcement policies (e.g., development of sanctuary communities, collaboration with ICE) and migrant families' actual and perceived access to resources (56). Thus, the advent of a deportation event triggers significant changes in the household and family structure, which can produce observable and measurable outcomes across diverse mental, physical, social and economic domains at the child, U.S.-based parent, and household levels. Finally, the framework proposes that the deportation process and event also affect the deportee's outcomes and the post-deportation circumstances of the deportee may have a reciprocal effect on the family and household remaining in the U.S. The well-being of migrants post-deportation has been examined in some diverse contexts (9, 18, 57–61) though the longitudinal and reciprocal relationships with their U.S.-based families have not received attention. This framework should be tested in qualitative and quantitative studies with diverse national origin migrant communities to better understand the impacts of deportation and family separation. Additionally, the framework should be further refined, if needed, to account for any additional impacts of maternal deportation.

## Discussion

This study describes the migration and deportation histories of Mexican male migrants who were deported to Mexico along with their perceptions about the impacts of their removal on their families, especially children, who remained in the U.S. This study provides a novel perspective on the consequences of deportation and family separation and we implemented a mixed methods study design and analyzed data collected from a large sample to achieve our aims. We contextualize our findings below within the broader literature.

Our data revealed that about two-thirds of study participants were long-term U.S. residents (i.e., 11+ years), echoing research and media reports released during the study time period, which recognized that many deportees were long-standing members of U.S. communities pre-deportation (62, 63). Regarding deportation histories, one-third of the full sample was reportedly not restricted from re-entering the U.S., though a similar proportion (31%) reported being banned from re-entering for ≥11 years, and some of these deportees reported a life-time ban, meaning they could never re-enter the country to re-unite with their families. The proportion of deportees who reported both being separated from their nuclear family and receiving a long-term ban (11+ years) was even greater (38%). These data suggest that the destabilization of the family unit due to deportation could not be remedied quickly by family reunification in the U.S. and thus, families remaining in the U.S. will be required to develop a complex strategies to overcome the adverse impacts of family separation resulting from deportation. Moreover, immigration policies treat migrants who re-enter the U.S. as criminals and migrants may be incarcerated if detected in the country after having received a ban (29). However, in a sample of Salvadoran fathers, 52.5% intended to return to the U.S. despite the possibility of incarceration (29). Longitudinal studies are needed to understand long-term family dynamics and related outcomes under diverse restricted entry conditions (e.g., 5 year ban, 10 year, lifetime ban, etc.).

The quantitative and qualitative data collected in our study illustrated the vast range of social, economic and mental health challenges faced by families remaining in the U.S., after the expulsion of a parent. Our data paint a picture of economic deprivation and vulnerability that has negative implications for the physical and mental well-being of the spouse/partner and children left behind. Findings suggest increased vulnerability to housing instability due to the loss of the deported migrants' income, and food insecurity and inability to meet the families' daily needs (e.g., clothing). Similar findings were observed in a qualitative study conducted with 125 Latino families residing in Los Angeles, illustrating the disproportionate harm and burden of male migrants' deportation on women and children (10). Moreover, Baker and Marchevsky noted that families remaining in the U.S. were unable to recuperate the earnings and financial contributions to the household made by the deported migrant, thus thrusting families into persistent economic disadvantage. Given the harsh anti-immigrant rhetoric that has persisted in the past decade, family members remaining in the U.S. may hesitate to apply for programs (e.g., Supplemental Nutritional Assistance Program) for which they are eligible for fear of future retributions by the government (10, 64). Studying the long-term financial strategies employed by families impacted by deportation requires leveraging mixed-methods approaches to account for not only measurable outcomes but decision-making processes used by those left behind.

The qualitative and quantitative data underscore the precarious position that children are placed in when they are forced to transition from a two-parent household to a single-parent household, a status that has been shown to be disadvantageous for many children (30, 45). It is particularly concerning that families remaining in the U.S. reported lacking regular and/or safe childcare following the deportation process, suggesting that children may be transitioning from a situation of supervision to limited or no supervision with a parent who is stretched emotionally, logistically and economically as they attempt to maintain a functioning household. These findings have been observed in other studies conducted with U.S.-based families that empirically demonstrated that immigration enforcement policies are likely to increase the likelihood of single parent households and especially households headed by married mothers whose spouses are absent (23, 65). As noted by Amuedo-Dorantes et al., ethical concerns regarding family-separation remain persistent issues given the “acknowledged importance of keeping families together for the sake of the children” (65).

Other implications for families include the potential disruption of children's academic trajectories following paternal deportation (10, 39, 62, 64); this outcome is particularly concerning because of its negative impact on immediate and future human capital development and future economic outcomes. For example, leaving school can place youths' futures at risk by potentially elevating the risk of precarity and disadvantage. We and others have observed women's changing roles due to men's deportation (10). It is unclear how mothers and fathers negotiate these evolving conditions within their partnership and in the context of parenting children of varied ages. Research on these topics is needed, particularly in light of the diverse reentry conditions stipulated by the government (e.g., short vs. long-term restrictions from reentry into the U.S.).

Finally, the qualitative data illustrated the complex and inter-related mental health consequences resulting from the deportation of a parent on their dependent children who remain in the U.S. Our findings echo those of other studies conducted in the U.S. with migrant families (23, 66). We learned that children experienced a range of mental health symptoms ranging from sadness and depression, to chronic crying, anger and resentment, and even a suicide attempt. Because of the deportation process, the father-child bond is weakened (23, 64). Specifically, children lack a physical relationship with the deported father, which means they are unable to receive the immediate and long-term verbal and non-verbal care and emotional support they need to develop into healthy, well-functioning, productive adults. Studies have documented how the absence of parental love and care can adversely impact the long-term well-being and development of children of incarcerated parents (67). Moreover, a growing body of research shows that Adverse Childhood Events, which include justice-system contact or parental incarceration before the age of 18, are associated with poor physical and mental health outcomes in adulthood (68) and other studies suggest that deportation events can be similarly traumatic to children (39, 43).

Deported parents consistently reported concerns about being unable to support their families emotionally and to provide the guidance and love that their children need. While a minority of parents in our study reported access to electronic means of communication (e.g., email, cell phone, internet café), these are likely insufficient to meet the complex emotional, physical and social needs of children throughout their formative years. More recent qualitative studies of deportees residing in Tijuana have illustrated the growing use of technology (e.g., video-conferencing) by separated families to maintain contact between deportees and families remaining in the U.S. (10). Efforts to promote communication among separated families are needed on both sides of the border to support children and partners remaining in the U.S. These efforts can help ensure that the parental-child and parent-parent bonds are reinforced over time.

While some families have relocated the entire family to Mexico in order to preserve the family unit, other studies have found that this process, including the relocation of U.S. citizen children to their parent's home country (e.g., Mexico), may be stressful and create new legal, social, mental health, and economic challenges (69). For example, some children may have never been to Mexico, or don't speak Spanish and given their lack of citizenship in the receiving country, may find themselves temporarily or permanently excluded from public institutions (e.g., state-sponsored schools, publicly funded health care systems) (69, 70). Qualitative studies suggest that relocating children of Mexican nationals (i.e., U.S.-born, and Mexican nationals raised in the U.S.) is an emotionally, economically, and socially complex process that merits coordination and preparation of the child (70, 71). For families who seek reunification in Mexico, it is important to consider strategies to socially integrate U.S.-born children of Mexican descent. Diverse approaches may be needed to reduce the resulting mental health and social impacts from such a relocation while also optimizing children's incorporation into academic institutions (71) so that they may advance their education to the benefit of their future well-being.

## Limitations

Our study has the following limitations; data were self-reported and may be subject to underreporting due to the sensitive nature of the topics. The sample was limited to males and Mexican nationals because few (*n* = 10) identified as Central American or women (*n* = 35) and these data could not support meaningful analyses for these subgroups. The findings may not be generalizable to other national origin groups. Participants were not asked to specify the exact ages of their children which prevents us from making specific observations regarding impacts by age. Future research should request the ages of children remaining in the U.S. so that developmentally sensitive conclusions can be made. Due to the sensitive nature of migration and deportation experiences, some participants may not have fully disclosed information. At the time of the study, extortions of U.S.-based family members were reported by participants. Therefore, participants may not have revealed a family separation in order to protect their family. We were also unable to corroborate the impact of the separation on family members remaining in the U.S., though findings are consistent with other published studies. We did not ask participants to report on the impact of their deportation on spouses or extended family members; our limited data suggest that these questions should be asked in future studies. Nevertheless, this mixed-methods study presents novel findings pertaining to the perceived impacts of family separation due to deportation with a large sample of male migrants, a group that may be easier to engage in research than the families left behind.

## Conclusion

Six years after these data were collected, the threat of or actual family separation events due to deportation remain significant factors challenging the well-being of immigrant families in the U.S. (50, 72). Therefore, strategies to reduce the adverse impacts of deportation events, especially among established migrants and those with deep ties to the U.S. (e.g., families with children), are needed. In Mexico, facilitating the social and economic reintegration of the deportee may help offset the economic burden of deportation on families in the U.S. who may shoulder the additional expense of maintaining a second household in Mexico for the deportee. For families seeking to reunite with the deportee in Mexico, ensuring that children have access to social (e.g., education) and other institutions (e.g., health care) is critical to promoting their well-being. It is critical that all parents and children have legal identification in the receiving country (e.g., Mexico), in order to prevent an undocumented status, which would prevent accessing public services (e.g., health care) and the labor market (e.g., employment opportunities) (61, 73).

On the U.S. side, reforming immigration policy is critically needed to address national security concerns while prioritizing the welfare of immigrant families in the U.S. Additional systematic and comprehensive research documenting the economic and social consequences of deportation and family separation is needed and can inform policy development and implementation. Families and children remaining in the U.S. would also benefit from comprehensive, trauma-informed, wrap-around interventions (74) to reduce the adverse mental health, psychosocial and economic outcomes resulting from forced family separation; evaluation of such interventions is especially needed. For example, one strategy may involve developing binational programs to improve family unity and parental involvement in the lives of children remaining in the U.S. The use of videoconferencing services may be a secondary approach if face-to-face contact is not possible (10, 75); however, there is a lack of data regarding the effect of this approach on children's and parent's mental well-being and child-parent and parent-parent relationships. Additionally, efforts to mitigate certain determinants of deportation (e.g., access to substance use rehabilitation programs, access to drivers' education programs and licenses) may promote the continued presence of two-parent households.

Finally, our proposed conceptual framework “A Framework for Researching the Outcomes of Family Separation Due to Paternal Deportation” suggests relationships, concepts and domains that can be tested in qualitative and quantitative studies in larger, nationally diverse samples and families experiencing maternal deportation. This framework intentionally recognizes the inter-relatedness of individual, community level and policy factors on diverse health and social outcomes. This approach may help foster creative solutions to address the myriad of challenges faced by immigrant families facing or living in a post-deportation context. However, there is a critical need to develop funding streams for research examining the determinants of well-being among U.S.-based mixed-status families and transnational families that include transnational migrants.

## Data Availability Statement

The datasets generated for this study are available on request to the corresponding author.

## Ethics Statement

The studies involving human participants were reviewed and approved by the University of California, San Diego Human Research Protection Program and the Ethics Board of the Clinic. The patients/participants provided their written informed consent to participate in this study.

## Author Contributions

VO and JB designed the study, oversaw data collection, conducted the analyses, interpreted the data, and prepared the manuscript. CM co-authored the manuscript, assisted with formatting, co-authored the framework, and provided feedback. AV-O assisted with interpreting the data and provided feedback on the interpretation of the data and manuscript.

### Conflict of Interest

The authors declare that the research was conducted in the absence of any commercial or financial relationships that could be construed as a potential conflict of interest.
